# Dynamics of Blood Lipids Before, During, and After Diurnal Fasting in Inactive Men: Quasi-Experimental Study

**DOI:** 10.2196/56207

**Published:** 2024-10-17

**Authors:** Khalid Aljaloud, Naif Al-Barha, Abeer Noman, Abdulaziz Aldayel, Yahya Alsharif, Ghareeb Alshuwaier

**Affiliations:** 1 Department of Exercise Physiology College of Sport Sciences and Physical Activity King Saud University Riyadh Saudi Arabia; 2 Department of Physical Activity Faculty of Education and Science Albaydha University Rada'a Yemen; 3 School of Physical Education and Sport Training Shanghai University of Sport Shanghai China; 4 Department of Food Science and Technology Faculty of Agriculture Sana’a University Sana’a Yemen

**Keywords:** cardiovascular diseases, cardiovascular risk factors, lipids, glucose measurement, fasting, Ramadan, body composition

## Abstract

**Background:**

There is a lack of investigation into the dynamics of blood lipids before, during, and after diurnal fasting, especially in inactive men.

**Objective:**

This study determined dynamic changes in blood lipids in inactive men before, during, and after they underwent diurnal fasting.

**Methods:**

A total of 44 young men aged a mean 27.6 (SD 5.8) years were recruited to evaluate their habitual physical activity and diet using a questionnaire developed for this study. Body composition was evaluated using a bioelectrical impedance analysis machine (Tanita BC-980). An 8-ml blood sample was collected to evaluate blood lipids and glucose. All measurements were taken 2-3 days before Ramadan, during Ramadan (at week 2 and week 3), and 1 month after Ramadan. A 1-way repeated measures ANOVA was used to compare the measured variables before, during, and after the month of Ramadan. When a significant difference was found, post hoc testing was used. Differences were considered significant at *P*<.05.

**Results:**

There was a significant reduction in low-density lipoprotein during Ramadan compared to before and after Ramadan (83.49 mg/dl at week 3 vs 93.11 mg/dl before Ramadan [*P*=.02] and 101.59 mg/dl after Ramadan [*P*=.007]). There were significant elevations in fasting blood glucose (74.60 mmol/L before Ramadan vs 81.52 mmol/L at week 3 [*P*=.03] and 86.51 mmol/L after Ramadan [*P*=.01]) and blood pressure (109 mm Hg before Ramadan vs 114 mm Hg after Ramadan; *P*=.02) reported during and even after the month of Ramadan, although both fasting blood glucose and blood pressure were within normal levels.

**Conclusions:**

Ramadan fasting could be an independent factor in reducing low-density lipoprotein. Further investigations are encouraged to clarify the impact of diurnal fasting on blood lipids in people with special conditions.

## Introduction

Ramadan is a lunar month that varies between 29 and 30 days. The world’s Muslim population is about 1.5 billion [1] and is expected to increase to 2.2 billion by 2030 [2]. Ramadan fasting is a form of religious diurnal fasting in which adult Muslims abstain from having food and drink (unless necessary) during the daylight hours, from dawn to sunset. The average diurnal fasting time during Ramadan month is 15 (SD 3) hours per day, depending on the season and the country’s geographical location. During the month of Ramadan, Muslims usually change their daily lifestyle [3,4]. Muslim adults usually become less active during Ramadan, especially in the daytime compared to the nighttime [5-7]. These changes, in addition to diurnal fasting, may negatively affect health-related blood biomarkers. Previous evidence concluded that metabolic disease biomarkers, including blood lipids, were elevated in women and urban residents [6]. Genetic factors are key in metabolic disease biomarkers, including high blood cholesterol, in different ethnic groups [8]. For example, some studies reported heritability of up to 70% for genes involving high-density lipoprotein (HDL) [9]. Diurnal fasting has been found to improve blood lipids [4,5,10,11].

The negative role of physical inactivity on blood lipids is evident. Physical inactivity has been found to play a key role in developing cardiovascular and metabolic disease risk factors such as obesity [12-15]. In a recent study conducted with young Saudi medical students, one of the most common risk factors of cardiovascular and metabolic disease was physical inactivity (57.9%) [16]. Insufficient physical activity is responsible for around 10% of all deaths globally, and sedentary behavior (SB) costs global health care organizations billions of dollars each year. Furthermore, the accumulation of low-density lipoprotein (LDL) cholesterol in artery walls causes narrowing of the arteries, which may lead to the development of cardiovascular disease (CVD) [17].

The number of studies investigating the association between Ramadan fasting and health promotion dramatically increased from 2010 to 2021, with 1276 studies being published [18]. Although there is robust available evidence exploring the impact of Ramadan fasting on health-related biomarkers, reports on the effect of diurnal fasting on blood lipids in inactive adults are scattered [5,11,18-20]. The impact of fasting during Ramadan on body composition and physiological parameters has been widely investigated [21-24]. During the month of Ramadan, people may achieve a significant reduction in body fat mass and blood lipids such as LDL. Some studies did not report significant improvement in triglyceride (TG) levels and fasting blood glucose (FBG) [23]. On the other hand, some blood and metabolic changes that occur during Ramadan fasting are temporary [21,25,26]. It seems that the positive change in LDL levels as a result of diurnal Ramadan fasting is one of these temporary changes [25]. The major changes in blood parameters observed in previous studies could have been due to the lifestyle of participants [22,24].

Despite the results of previous studies, there are few recent studies that investigated the impact of fasting in the month of Ramadan (this fasting lasts about 15 hours during the daytime) during the summer season, where temperatures reach a mean 45 ºC (SD 3 ºC) during the daytime and about 35 ºC (SD 5 ºC) during the night. Thus, the main aim of this study was to investigate the dynamics of blood lipids before, during, and after diurnal fasting in inactive men in hot environments.

## Methods

### Participants

This quasi-experimental pre-post design study was conducted at 4 different time points (before Ramadan, at week 2 and week 3 during Ramadan, and after Ramadan). A total of 44 inactive male students (mean age 27.6, SD 5.8 years) were recruited to participate in this study. Inclusion criteria were being male, an adult (aged 18-39 years), and inactive, that is, performing less than the recommended level of physical activity (PA) for adults (150 min of moderate PA per week, 75 min of vigorous PA per week, or an equivalent amount of moderate-to-vigorous PA per week). Smokers or individuals taking medicines that may have affected the results were excluded from participating in this study. The sample size was calculated based on the change in LDL concentration reported in a previous study [27] with the 95% CI and 80% power for the test. The estimated sample size was 38 participants. The number of recruited participants was increased by about 10% to prevent the results being affected by withdrawals or incomplete data; thus, 44 apparently healthy inactive male students were recruited to participate in this study.

### Ethical Considerations

A consent form was read and signed by each participant before taking part in this study. Ethical approval was obtained from the Deanship of Graduate Studies and Scientific Research at King Saud University (CSSPA-22-07-35). The participants did not receive any compensation. All data were saved anonymously in a safe and confidential format by the principal investigator.

### Procedures

Each participant was instructed by the principle investigator to visit the laboratory on the same day of the week on all 4 occasions. The measured variables were obtained at each visit. The first visit was 2 to 3 days before the month of Ramadan, and the second and third visits were in the second week and third week of Ramadan. The fourth visit took place 6 weeks after the month of Ramadan. The procedures of this study were performed consistently as described in the following sections.

### Physical Characteristics

Body mass (BM), height, and waist circumference (WC) were measured. BMI was calculated by dividing body weight in kilograms by height in meters squared (kg/m^2^). Fat-free mass (FFM), body fat (BF) percentage, and total body water (TBW) percentage were evaluated using bioelectrical impedance analysis (BC-418; Tanita) [28].

### PA and Diet Assessment

PA and diet behavior were evaluated using a valid and reliable self-reported questionnaire for the assessment of PA and diet behavior in youth aged 15 to 25 years [29]. Each participant was instructed on how to fill out the PA and diet questionnaire. The questionnaire provided continuous variables (time in minutes). The average time to complete the questionnaire was 10 minutes. The diet behavior section assessed the type and frequency of the consumed food.

### Physiological and Lipid Profile

Resting heart rate (HR) and blood pressure (BP) were recorded following 10 minutes of being seated on a chair. All participants were instructed to fast at least 10 hours before attending the laboratory for blood collection. A venous blood sample of 8 ml was drawn by a phlebotomist to evaluate blood lipids, including TG, total cholesterol (TC), HDL, and LDL. Blood samples were analyzed in a specialized medical laboratory at the university.

### Statistical Analysis

Data from this study were analyzed using SPSS (version 24.0; IBM Corp). Results were illustrated as means with SDs. A 1-way repeated measures ANOVA was used to compare the measured variables before, during, and after the month of Ramadan. The Tukey post hoc test was used when a significant difference was found. The *χ*^2^ test was used to determine the statistical significance of associations between food consumed per week for participants across all trials. Results were considered statistically significant at *P*<.05.

## Results

The main aim of this study was to investigate the dynamics of blood lipids before, during, and after diurnal fasting in inactive men. Table 1 shows the physical characteristics of the participants before, during, and after Ramadan. This study did not observe significant changes in any of the body composition variables, including BM, BMI, WC, BF mass, BF percentage, FFM, FFM percentage, and TBW between all measured occasions.

**Table 1:** Measurements of body composition at the 4 time points (N=44).

Body composition measurements	Before Ramadan, mean (SD)	Ramadan week 2, mean (SD)	Ramadan week 3, mean (SD)	After Ramadan, mean (SD)	*F* test (*df*)	*P* value
Body mass (kg)	70.0 (12.6)	69.6 (12.4)	69.6 (12.8)	69.9 (12.9)	0.03 (43)	.99
BMI (kg/m^2^)	24.5 (4.0)	24.3 (4.0)	24.3 (3.8)	24.3 (4.0)	0.04 (43)	.99
Waist circumference (cm)	82.9 (10.9)	82.3 (10.6)	81.8 (10.5)	81.8 (11.0)	0. 10 (43)	.96
Body fat mass (kg)	14.7 (6.6)	14.0 (6.9)	13.9 (6.8)	14.5 (6.9)	0.13 (43)	.94
Body fat percentage (%)	20.2 (6.2)	19.5 (6.7)	19.4 (6.6)	19.7 (6.5)	0.22 (43)	.88
Fat-free mass (kg)	55.3 (8.6)	55.6 (6.9)	55.5 (7.5)	55.8 (7.3)	0.04 (43)	.99
Fat-free mass percentage (%)	79.8 (6.2)	80.5 (9.0)	80.6 (6.7)	80.3 (6.5)	0.13 (43)	.94
Total body water (liters)	40.1 (6.0)	41.6 (6.8)	40.5 (5.8)	40.6 (5.4)	0.49 (43)	.69
Total body water percentage (%)	56.9 (7.2)	59.0 (5.0)	59.3 (5.3)	58.9 (5.0)	1.56 (43)	.20

### Physical Activity

Based on the PA guidelines (moderate-to-vigorous PA ≥150 min/week for ≥5 days/week or ≥70 min/week for ≥3 days/week) [30], participants were physically inactive. Moreover, there was no significant change in PA level across the 4 time points (99.8, SD 210.9; 125.7, SD 260.4; 111.0, SD 275.4; and 56.7, SD 151.0 minutes before Ramadan, in the second and third weeks of Ramadan, and after Ramadan, respectively; Table 2). The average sedentary time of the participants was less than 6 hours (ranging from 5.2, SD 1.9 to 5.8, SD 2.2 h/day) across all 4 time points.

**Table 2:** Physical activity levels across all 4 time points (before Ramadan, in the second and third weeks of Ramadan, and after Ramadan; N=44).

Physical activity and sleeping time	Before Ramadan, mean (SD)	Ramadan week 2, mean (SD)	Ramadan week 3, mean (SD)	After Ramadan, mean (SD)	*F* test (*df*)	*P* value
Moderate activity (min/wk)	50.9 (121.7)	64.4 (137.6)	50.2 (154.51)	34.6 (89.5)	0.40 (43)	.75
Vigorous activity (min/wk)	48.9 (108.4)	61.3 (143.9)	60.8 (162.4)	22.2 (76.0)	0.92 (43)	.43
Moderate + vigorous (min/wk)	99.8 (210.9)	125.7 (260.4)	111.0 (275.4)	56.7 (151.0)	0.74 (43)	.53
Sedentary behavior (h/day)	5.8 (2.2)	5.2 (1. 9)	5.7 (3.0)	5.5 (2.6)	0.56 (43)	.64
Sleeping duration (h/day)	6.9 (1.0)	6.6 (1.1)	6.7 (1.0)	6.3 (1.1)	2.58 (43)	.06

### Diet Behavior

Diet behavior was reported by evaluating types and frequencies of consumed food. Diet behavior is one of the essential factors that could have impacted this study’s outcomes. Thus, participants’ diet behavior was evaluated across all 4 time points. Table 3 demonstrates the consumed food per week at all 4 time points. The frequency of the consumed food was classified into 3 categories: no more than twice per week, 3 to 5 times per week, and ≥6 times per week. There were no significant changes in the frequency of consumed food per week across the 4 time points.

**Table 3:** Percentage of consumed food per week for participants across all 4 time points (before Ramadan, in the second and third weeks of Ramadan, and after Ramadan; N=44).

Food types	Frequency of consumed food per week (participants), n (%)				Chi-square (*df*)		*P* value
	<2 times/w	<3-5 times/w	>6 times/w				
Vegetables	12 (28)	17 (40)	14 (32)	3.97 (43)		.68	
Fruit	9 (21)	21 (48)	14 (32)	9.11 (43)		.17	
Milk and dairy products	13 (30)	18 (40)	13 (30)	5.62 (43)		.47	
Soda and soft drinks	19 (44)	15 (34)	10 (22)	11.37 (43)		.08	
Energy drinks	39 (89)	4 (10)	2 (1)	3.90 (43)		.27	
Fast food	19 (43)	18 (43)	6 (14)	6.20 (43)		.40	
Chips and french fries	20 (46)	17 (40)	6 (14)	3.70 (43)		.72	
Biscuits and cake	24 (55)	11 (26)	9 (20)	11.97 (43)		.22	
Chocolate and sweets	16 (36)	13 (31)	14 (33)	9.30 (43)		.16	

### Physiological and Blood Parameters

**Figure 1:**
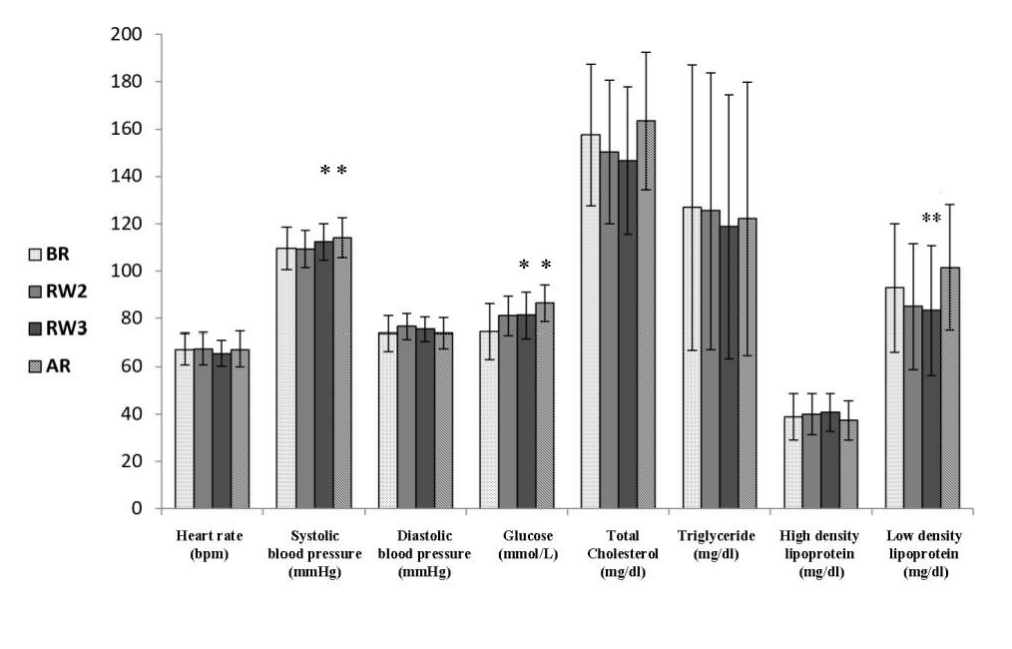
The effect of Ramadan fasting on resting heart rate (HR), systolic blood pressure, diastolic blood pressure, glucose, total cholesterol, triglyceride, high density lipoprotein (HDL) and low-density lipoprotein (LDL), (n=44).

Physiological and blood parameters, including resting HR, BP, and blood lipids, were analyzed. The results of this study showed a significant effect of diurnal fasting on both FBG and LDL (Figure 1). Furthermore, a significant elevation was recorded in systolic blood pressure (SBP) 1 month after Ramadan compared to before Ramadan (109 mm Hg before Ramadan vs 114 mm Hg after Ramadan). Similarly, FBG was slightly elevated during week 3 of Ramadan and after Ramadan compared to before Ramadan (74.60 mmol/L before Ramadan, 81.52 mmol/L at week 3, and 86.51 mmol/L after Ramadan). However, the results showed an obvious reduction in LDL during week 3 of Ramadan compared to before and after Ramadan month (83.49 mg/dl at week 3 vs 93.11 mg/dl before Ramadan [*P*=.02] and 101.59 mg/dl after Ramadan [*P*=.007]).

## Discussion

### Principal Findings

The impact of diurnal fasting during the month of Ramadan on blood lipids in inactive men was limited but positive and very obvious for LDL. Although there were no significant changes in PA or SB across all trials (*P*=.53 for moderate-to-vigorous PA; *P*=.64 for SB), some blood lipids, such as LDL, may be affected positively during fasting in the month of Ramadan. The novelty of this study was controlling for the main related factors that may affect blood lipid variables. In contrast, this study did not find significant changes in the participants’ diet behavior across the investigated time points (all *P*>.05). In general, the findings of this study may help inactive individuals control blood lipids, especially LDL, while fasting diurnally.

### Body Composition

All body composition variables investigated in this study did not change significantly across any of the 4 time points. The novelty of this study was examining the impact of Ramadan fasting in a hot and dry environment (≥45 ºC) on blood lipids. In this study, body mass, BMI, and WC decreased gradually, especially during the third week of Ramadan, compared to before Ramadan. However, the recorded reduction was not significant (*P*=.88 for BF percentage, *P*=.94 for FFM percentage, *P*=.20 for BW percentage, *P*=.99 for both BM and BMI, and *P*=.96 for WC). While some previous studies have reported significant decreases in body composition parameters at the end of Ramadan compared to measurements taken before Ramadan [21], numerous previous studies have concurred with the findings of this study [7,25,26,31-33]. In fact, the results of this study are reasonable, as most of the participants were within normal weight. However, some studies have reported a reduction in BW due to a decrease in skeletal muscle mass and FFM [34]. Furthermore, the significant reduction in body composition in the previous literature may be linked to the obesity status of the participants [35]. The majority of the participants in this study were within normal body composition measurements. Therefore, the impact of Ramadan fasting may not influence body composition in individuals with normal body composition [36]. Focusing on apparently healthy men could be one of the limitations of this study. Further studies in different ages and genders are encouraged to understand the impact of diurnal fasting on body composition parameters.

### Physiological and Blood Parameters

The results for both SBP and FBG were elevated significantly during the month of Ramadan (*P*=.02 and *P*=.01, respectively), especially during the end of Ramadan (week 3) compared to levels before Ramadan. These findings concur with previous studies [22,37]. However, the elevated values of SBP and FBG were within the healthy range (SBP: 109 mm Hg before Ramadan vs 114 mm Hg after Ramadan; FBG: 74.60 mmol/L before Ramadan vs 81.52 mmol/L at week 3 and 86.51 mmol/L after Ramadan). One novel aspect of this study was the impact of diurnal fasting on LDL, as there were no significant changes in the main factors that may impact LDL concentration, such as body composition, PA, and diet behavior. Thus, diurnal fasting itself could be an independent factor that positively improved LDL. Our findings concur with the results of a previous study that investigated the impact of Ramadan fasting on college students [23,36,38]. However, it seems that the observed positive, significant change in LDL concentration as a result of diurnal fasting during the month of Ramadan was temporary [25,38]. The novelty of this study is its examination of the impact of diurnal fasting on blood lipids in inactive men with a stable lifestyle. Blood biomarkers, including blood lipid concentration, may be positively improved by diurnal fasting. However, this study discovered that the changes in blood lipid variables were limited in healthy but inactive young men. The major changes in blood parameters observed in previous studies may refer to the nature of participants’ lifestyles [22,24,39]. Further investigations are encouraged to clarify the impact of diurnal fasting on blood lipids in people with special conditions, such as older age and obesity.

This study has some limitations. First, it was conducted with apparently healthy young adult men. Second, physical activity was not assessed by an objective measurement such as an accelerometer. Third, some other confounders were not evaluated, such as the total energy expenditure and basal metabolic rate of the participants. Moreover, the sample size of the study may not have been enough to detect differences between the time points.

### Conclusion

Diurnal intermittent fasting seems to be an independent factor that could improve blood lipids, including LDL, even in healthy but inactive adults. Moreover, diurnal fasting has been found to decrease LDL even in inactive adults with normal LDL levels. However, the results of this study concur with previous studies in terms of the temporary effect of diurnal fasting during the month of Ramadan on blood lipids. More research is encouraged to examine the impact of Ramadan fasting in people with special health conditions, such as obesity or older age.

## Abbreviations

BF: body fat
BM: body mass
BP: blood pressure
CVD: cardiovascular disease
FBG: fasting blood glucose
FFM: fat-free mass
HDL: high-density lipoprotein
HR: heart rate
LDL: low-density lipoprotein
PA: physical activity
SB: sedentary behavior
SBP: systolic blood pressure
TBW: total body water
TC: total cholesterol
TG: triglyceride
WC: waist circumference

## Data Availability

The data sets generated during this study are available from the corresponding author on reasonable request.
